# An effective frequency-domain feature of atrial fibrillation based on time–frequency analysis

**DOI:** 10.1186/s12911-020-01337-1

**Published:** 2020-11-25

**Authors:** Yusong Hu, Yantao Zhao, Jihong Liu, Jin Pang, Chen Zhang, Peizhe Li

**Affiliations:** College of Information Science and Engineering, Northeastern University, Shenyang, Liaoning China

**Keywords:** Atrial fibrillation, Frequency-domain feature, Time–frequency analysis, ECG, Decision tree algorithm

## Abstract

**Background:**

Atrial fibrillation is a type of persistent arrhythmia that can lead to serious complications. Therefore, accurate and quick detection of atrial fibrillation by surface electrocardiogram has great importance on further treatment. The practical electrocardiogram signals contain various interferences in different frequencies, such as myoelectricity interference, power interference and so on. Detection speed and accuracy largely depend on the atrial fibrillation signal features extracted by the algorithm. But some of the discovered atrial fibrillation features are not well distinguishable, resulting in poor classification effect.

**Methods:**

This paper proposed a high distinguishable frequency feature—the frequency corresponding to the maximum amplitude in the frequency spectrum. We used the R–R interval detection method optimized with the mathematical morphology method and combined with the wavelet transform method for analysis. According to the two features—the maximum amplitude in the frequency spectrum and R–R interval irregular, we could recognize atrial fibrillation signals in electrocardiogram signals by decision tree classification algorithm.

**Results:**

The data used in the experiment come from the MIT-BIH database, which is publicly accessible via the web and with ethical approval and consent. Based on the input of time-domain and frequency-domain features, we classified sinus rhythm signals and AF signals using the decision tree generated by classification and regression tree (CART) algorithm. From the confusion matrix, we got the accuracy was 98.9%, sensitivity was 97.93% and specificity was 99.63%.

**Conclusions:**

The experimental results can prove the validity of the maximum amplitude in the frequency spectrum and the practicability and accuracy of the detection method, which applied this frequency-domain feature. Through the detection method, we obtained good accuracy of classifying sinus rhythm signals and atrial fibrillation signals. And the sensitivity and specificity of our method were pretty good by comparison with other studies.

## Background

Atrial fibrillation (AF) is the most common arrhythmia, with a prevalence rate of 1.5% to 2% in developed countries [1]. When AF occurs, the regular order of atrial electrical activity disappears, replaced by the fast and disorderly tremor waves, and the atrial electrical activity is seriously disordered. Patients with AF are often accompanied by symptoms such as palpitations, arrhythmia, shortness of breath, and chest pain. The incidence of AF increases with age, and the most serious complication is stroke. Early diagnosis can effectively reduce the incidence of complications caused by AF.

**Fig. 1.**
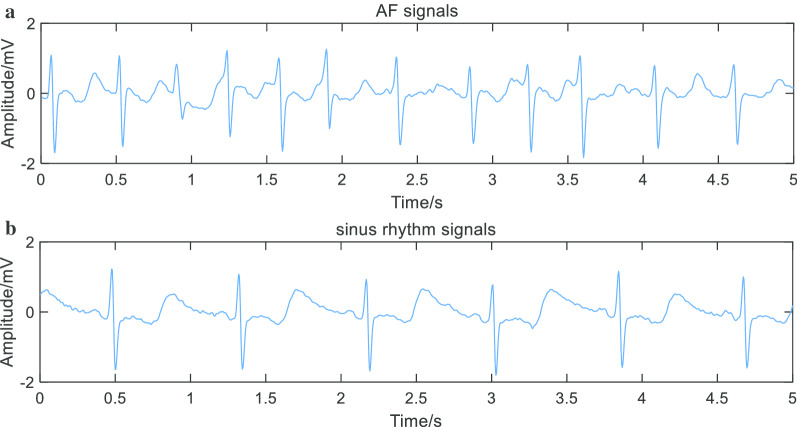
5 s original ECG signal in AF (**a**) and sinus rhythm (**b**). It can be seen that P waves are replaced by irregular F waves in the AF signals. Other waves are not very different between the AF signal and sinus rhythm signal

An electrocardiogram (ECG) is a technique that uses a medical device to collect and record a pattern of changes in activity produced by the heart. Compared with other bioelectrical signals, ECG signals are easier to monitor and have morphological regularity. Typical ECG signals mainly include P wave, Q wave, R wave, S wave, and T wave, as shown in Fig. 1. When AF occurs, the original normal P-waves disappear and are replaced by a series of irregular high-frequency oscillations called F-waves; the distance between R waves varies irregularly. The above two features have become the basis of the current automatic detection AF technology [2].

The current diagnosis of AF relies primarily on the presence of some typical symptoms of the patient and the characteristics of the ECG recording. However, early and accurate detection of AF remains a challenge. The detection of asymptomatic paroxysmal AF needs about 72-h ECG signals [3]. Therefore, it is valuable to develop an automatic detection algorithm that can diagnose AF quickly, accurately and reliably [2]. It is also of great significance to explore effective and high distinguishable features of atrial fibrillation to realize the automatic detection of atrial fibrillation.

Moody et al. proposed an automatic method for detecting AF based on the difference between the AF signal and the sinus rhythm signal in the RR interval [4]. Tateno et al. proposed a method based on the coefficient of variation and density histograms of RR and ΔRR intervals [5]. They identify the difference between sinus rhythm signal and AF signal by using the Kolmogorov–Smirnov test. These studies based on the RR interval achieved 97% accuracy of automatic detection. Using empirical mode decomposition, Uday Maji et al. found significant differences in the fourth layer intrinsic mode function (IMF4), with an accuracy of 96% [6].

Recently, some scholars have regarded AF as an abnormal phenomenon and analyzed it as a signal abnormality. Paolo Massimo Buscema et al. [7] proposed to apply an improved Back Propagation neural network for the diagnosis of AF. This method used a Supervised Contractive Map neural network structure and achieved the diagnosis of AF with an accuracy rate of 95%. He Runnan et al. [8] proposed a way of detecting AF based on Continuous Wavelet Transform(CWT) and two-dimensional convolutional neural network by analyzing ECG’s overall time–frequency features. Asgari et al. [9] applied wavelet transform to extract peak-to-average power ratio and logarithmic energy entropy as feature vectors for AF detection.

Common methods to extract F wave include the QRST cancellation method, ICA analysis method based on principal component analysis, etc. The QRST cancellation method is very sensitive to the change of waveform and greatly depends on the quality of F-wave extraction. The method in this paper focused on the ECG signals’ frequency-domain feature. By analyzing the decomposition results of each layer of the wavelet transform, we got an effective frequency-domain feature and used the feature as one of the bases for detecting AF. This method did not depend on the extraction of F waves. Simultaneously, our detection results had good accuracy, sensitivity, and specificity.

## Methods

### Outline of ECG processing methods

**Fig. 2:**
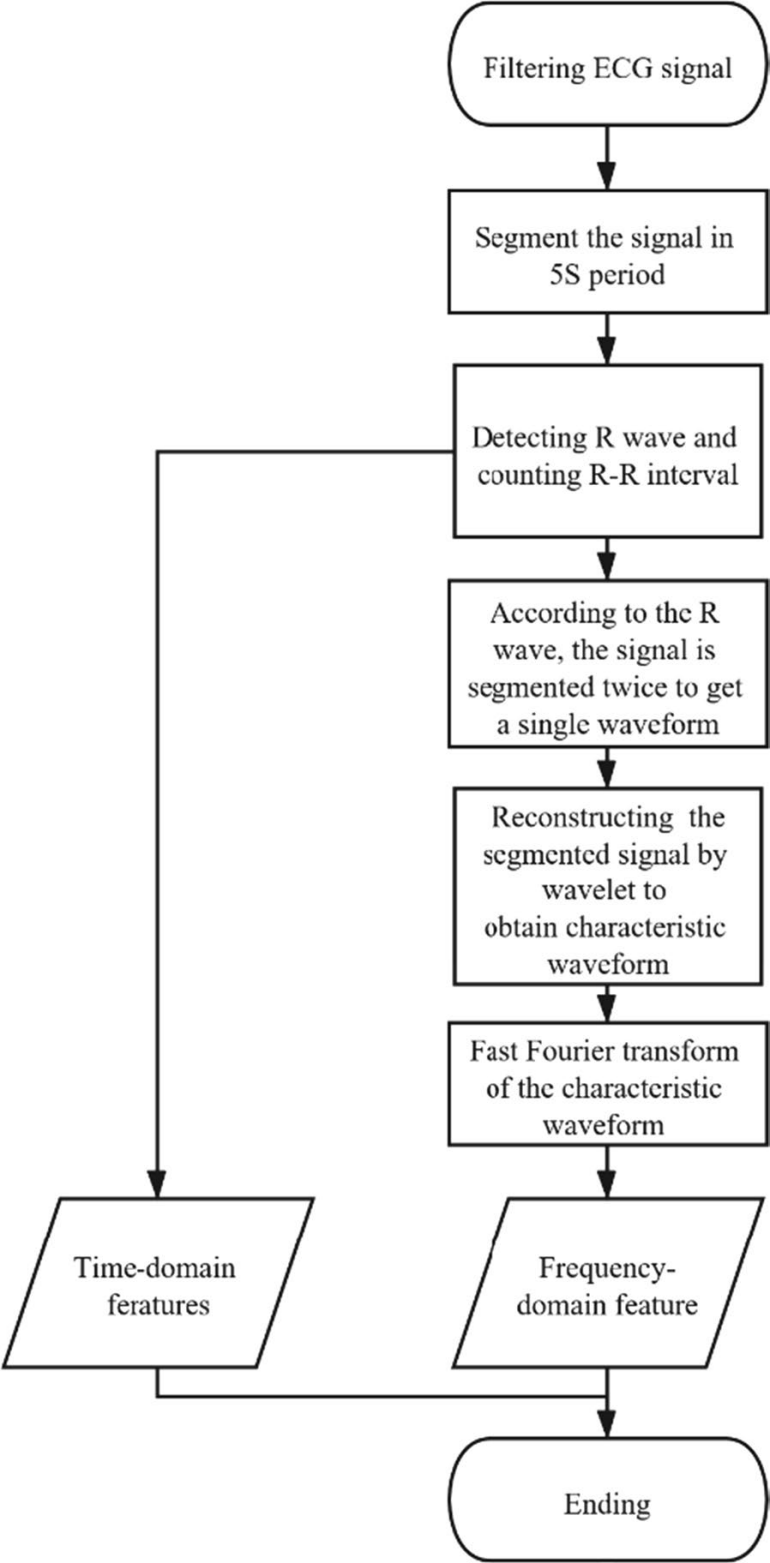
Procedures of extracting features

First of all, we removed the high-frequency noise and baseline drift of the ECG signal by filtering. Then the ECG signal was segmented by 5 s to detect the R wave peaks of each period. In this way, we could extract the mean and variance of the R–R interval, which could identify the degree of regularity of the R–R interval and obtain the time domain characteristics of the ECG signals. Next, the filtered signal was segmented according to R peak to obtain a single-period signal waveform. Then we decomposed the single-period signal waveform by wavelet transform. And we reconstructed the characteristic waveform by the approximate decomposition coefficients of the fourth layer. Furthermore, we obtained the frequency corresponding to the maximum amplitude in the frequency spectrum (MAiFS) by fast Fourier transform of the characteristic waveform. Thus we gained the frequency domain feature of the ECG signals. The above two types of features were used as the finally extracted AF signal features. And using the decision tree classification algorithm to detect AF. Finally, we proved the validity of the extracted frequency-domain features and obtained the accuracy, sensitivity, and specificity of the detection method of AF through the MIT-BIH AF dataset. The processes of the method are shown in Fig. 2.

### Time-domain features extraction method

#### Mathematical morphology filtering

Mathematical morphology [10] is an image analysis discipline based on lattice theory and topology. The basic operations include corrosion and expansion.

Let ${\text{f}}\left( {\text{n}} \right),\left( {{\text{n}} = 0,1,...,{\text{N}} - 1} \right)$ and ${\text{g}}\left( {\text{m}} \right),{ }\left( {{\text{m}} = 0,1, \ldots ,{\text{M}} - 1} \right)$, among them ${\text{N}} \gg {\text{M}}$. ${\text{g}}\left( {\text{m}} \right)$ is the structural element of the morphological filter. The selection of ${\text{g}}\left( {\text{m}} \right)$ should be similar to the shape of the preserved waveform and different from the shape of the filtered waveform. To preserve the R-wave and filter out other waveforms, we chose the structural element ${\text{g}}\left( {\text{m}} \right) = \left\{ {1,1,1} \right\}$.

Corrosion operation is defined as

$$\left( {{\text{f}}\Theta {\text{g}}} \right)\left( {\text{n}} \right) = \mathop {{\text{min}}}\limits_{{{\text{m}} = 0,1, \ldots ,{\text{M}} - 1}} \left\{ {{\text{f}}\left( {{\text{n}} + {\text{m}}} \right) - {\text{g}}\left( {\text{m}} \right)} \right\}$$

Expansion operation is defined as

$$\left( {{\text{f}} \oplus {\text{g}}} \right)\left( {\text{n}} \right) = \mathop {{\text{max}}}\limits_{{{\text{m}} = 0,1, \ldots ,{\text{M}} - 1}} \left\{ {{\text{f}}\left( {{\text{n}} - {\text{m}}} \right) + {\text{g}}\left( {\text{m}} \right)} \right\}$$

Because of corrosion operation and expansion operation have time sequence, mathematical morphology gives two different morphological operations. Corrosion first followed by expansion is defined as an open operation and expansion first followed by corrosion as a closed operation. Defining ${\text{f}}({\text{n}})$ on ${\text{g}}({\text{n}})$ open operation

1 $${\text{f}} \circ {\text{g}} = \left( {{\text{f}}\Theta {\text{g}}} \right) \oplus {\text{g}}$$

${\text{f}}({\text{n}})$ on ${\text{g}}({\text{n}})$ closed operation is defined as

2 $${\text{f}} \bullet {\text{g}} = \left( {{\text{f}} \oplus {\text{g}}} \right){\Theta g}$$

Through mathematical analysis, it can be proved that the morphological opening operation can flatten the peak and the closed operation can fill the trough. For ECG signals, the waveforms except the R wave can be flattened by the mathematical morphology operation.

#### Shannon energy envelope

Considering that the ECG signal fluctuates greatly near the R wave and according to the Shannon energy function [11], the response to the low amplitude is weak in the range of (0,1), and the response to the high amplitude is strong. We performed derivative and normalization on the filtered signal. Then the resulting function values were smoothly enveloped by a moving average method. The range of (0,1) means the normalized amplitude and is unitless.

${\text{d}}\left( {\text{n}} \right)$ is the derivative of the ECG signal. The Shannon energy operation is defined as

3 $$y_{1} \left( {\text{n}} \right) = - \left| {{\text{d}}\left( {\text{n}} \right)} \right|^{2} \times \ln (\left| {{\text{d}}\left( {\text{n}} \right)} \right|^{2} )$$

To prevent signal signature delays during smoothing, we used a sliding mean filter without phase shift

4 $${\text{y}}\left( {\text{n}} \right) = \frac{1}{{\text{N}}}\left( {y_{1} \left( {{\text{n}} - \frac{{{\text{N}} - 1}}{2}} \right) + y_{1} \left( {{\text{n}} - \frac{{{\text{N}} - 1}}{2} + 1} \right) + \cdots + y_{1} \left( {{\text{n}} + \frac{{{\text{N}} - 1}}{2}} \right)} \right)$$

If window overflow occurs in the head or tail segment of the signal, making $\min (1,{\text{n}} - \frac{{{\text{N}} - 1}}{2})$ and $\max ({\text{L}}\left( {{\text{signal}}} \right),{\text{n}} + \frac{{{\text{N}} - 1}}{2})$ do some appropriate changes. The N in the denominator of the formula should be appropriately adjusted. L is the length of the signal.

Through the Shannon energy envelope, we obtained the specific position of R peak. Simultaneously, the refractory period is set after each R peak detection. In the refractory period, even if there is a peak in the signal, it is not considered to be an R peak. In this test model, the refractory period was set to 200 ms.

### Frequency domain feature extraction method

Wavelet transform (WT) [12] is a powerful technology for representing a signal in different translations and scales. In practical applications, since the ECG signal is a short-term non-stationary random process, the Fourier transform based on the stationary stochastic process cannot reflect the essential characteristics of AF. The wavelet transform analysis method provides the possibility of extracting non-stationary random signal features.

#### Wavelet transform theory

For any signal $f(t) \in L^{2} (T)$, the wavelet transform is

5 $${\text{W}}_{{\text{f}}} ({\text{a}},{\text{b}}) = \left\langle {{\text{f}},\psi_{{{\text{a}},{\text{b}}}} } \right\rangle = |{\text{a}}|^{{ - \frac{1}{2}}} \mathop \int \limits_{{\text{R}}} {\text{f}}({\text{t}})\overline{{\psi \left( {\frac{{{\text{t}} - {\text{b}}}}{{\text{a}}}} \right)}} {\text{dt}}$$

where $\psi ({\text{t}})$ is a mother wavelet, ${\text{a}}$ is the dilation factor and b is the translation factor. Different frequency and time localizations can be achieved by adjusting a and b.

Since the ECG signal is stored in the form of discrete finite-length signals, continuous wavelet changes must be discretized for ease of calculation. Usually, the discrete formula of the dilation factor and the translation factor in the continuous wavelet transform is taken as: ${\text{a}} = {\text{a}}_{0}^{{\text{m}}}$, ${\text{b}} = {\text{na}}_{0}^{{\text{m}}} {\text{b}}_{0}$, where ${\text{m}},{\text{n}} \in {\text{Z}}$, ${\text{a}}_{0} \ne 1$. The corresponding discrete wavelet function can be expressed as

6 $$\psi_{{{\text{m}},{\text{n}}}} \left( {\text{t}} \right) = {\text{a}}_{0}^{{ - \frac{{\text{m}}}{2}}} \psi \left( {\frac{{{\text{t}} - {\text{na}}_{0}^{{\text{m}}} {\text{b}}_{0} }}{{{\text{a}}_{0}^{{\text{m}}} }}} \right) = {\text{a}}_{0}^{{ - \frac{{\text{m}}}{2}}} \psi \left( {{\text{a}}_{0}^{{ - {\text{m}}}} {\text{t}} - {\text{nb}}_{0} } \right)$$

At this point, the discrete wavelet transform of f(t) is

7 $${\text{WT}}_{{\text{f}}} \left( {{\text{m}},{\text{n}}} \right) = \mathop \int \limits_{{\text{R}}} {\text{f}}\left( {\text{t}} \right)\overline{{\psi_{{{\text{m}},{\text{n}}}} \left( {\text{t}} \right)}} {\text{dt}}$$

Its reconstruction formula is

8 $${\text{f}}\left( {\text{t}} \right) = {\text{C}}\mathop \sum \limits_{ - \infty }^{\infty } \mathop \sum \limits_{ - \infty }^{\infty } {\text{WT}}_{{\text{f}}} \left( {{\text{m}},{\text{n}}} \right)\psi_{{{\text{m}},{\text{n}}}} \left( {\text{t}} \right)$$

#### Mallat algorithm

Multi-resolution analysis constructs a series of orthogonal function spaces to decompose the sequence into a low-frequency signal and a series of high-frequency signals (the number of high-frequency signals depends on the number of decomposition layers). As for discrete-time signals, the dyadic discrete wavelet transform (DWT) can be implemented by low-pass, h(n), and high-pass, g(n), filters [13]. The Mallat algorithm is a fast algorithm for constructing orthogonal wavelets. The recursive formula of the decomposition can be expressed as

$$\begin{array}{*{20}c} {{\text{CA}}_{{{\text{j}} + 1}} = {\text{H*CA}}_{{\text{j}}} } \\ {{\text{CD}}_{{{\text{j}} + 1}} = {\text{G*CD}}_{{\text{j}}} } \\ \end{array}$$

where ${\text{CA}}_{{\text{j}}}$ and ${\text{CD}}_{{\text{j}}}$ are respectively column vector forms of wavelet coefficients, and H and G are respectively a matrix composed of low-pass filtering and high-pass filter coefficients of the corresponding filter. j is the number of decomposition layers of the wavelet transform.

The signal reconstruction process can be expressed as

9 $${\text{CA}}_{{\text{j}}} = {\text{H}}^{*} {\text{CA}}_{{{\text{j}} + 1}} + {\text{G}}^{*} {\text{CD}}_{{{\text{j}} + 1}}$$

It can be seen that the essence of the wavelet transform is a filtering process. The obtained approximate coefficients represent the low-frequency characteristics of the signal, and the detail coefficients represent the high-frequency characteristics of the signal. Through the wavelet transform, we can focus on the frequency characteristics of a certain frequency band of the ECG signal. We decomposed the ECG signal by wavelet, and reconstruct signals by using the data of each frequency band after decomposition. Then we analyzed the frequency domain characteristics of ECG signals by reconstructed signals. Therefore, the wavelet transform can be used to analyze the ECG signal and extract the frequency domain features of AF.

## Results

### Data source and preprocessing

**Fig. 3:**
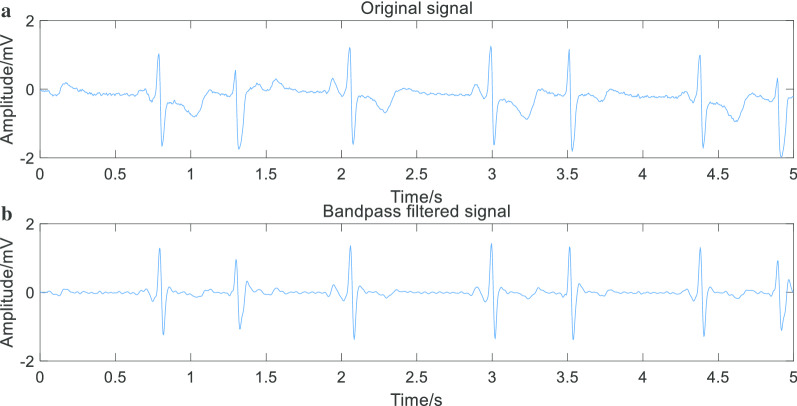
Comparation of original filtered signal and band pass filtered signal. The images show that original signal have some kinds of frequency interference and the band pass filtered signal is more regular than original signal

The data used in the experiment comes from the MIT-BIH database [4], which is publicly accessible via the web and with ethical approval and consent. The dataset contains 23 annotated ECG records, each of which is approximately 10 h with a sampling rate of 250 Hz and a 12-bit resolution with a range of 10mv. Each record contains two signals, ECG1 and ECG2. In this study, we used ECG1 to do these experiments. The preprocessing was divided into two steps: splitting the signal and filtering. The splitting signal was to divide the input ECG signal into segments of 5 s for subsequent processing. Filtering was to design an FIR digital filter by using a window function method and filtering the ECG signal. Its cutoff frequency was set to 0.5 Hz and 30 Hz. The purpose of setting a cutoff frequency to 30 Hz was to eliminate electromyography interference and 50 Hz frequency interference. The purpose of setting a cutoff frequency of 0.5 Hz was to eliminate human respiration, movement of the electrode and other low-frequency interference. The results were shown in Fig. 3.

### Time-domain feature extraction

As the mean and variance of R–R interval can represent the regularity of ECG signal in different conditions, the mean and variance of R–R interval in sinus rhythm and AF were taken as time-domain features in this paper. The processes can be divided into three steps: mathematical morphological filtering, determining the R-wave position by using the fragrance energy envelope, extracting R-wave waveform and analyzing time-domain features.

**Fig. 4:**
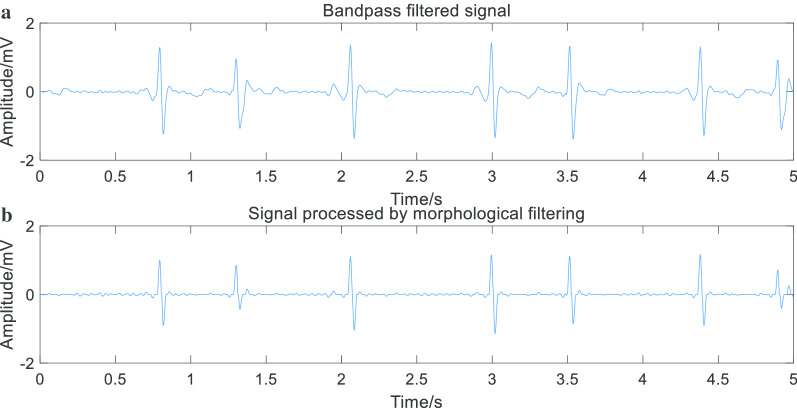
Compare band-pass filtered signal with morphological filtered signal. The image shows that the morphological filter can further eliminate interferences than band-pass filter so that we can obtain the needful signal to do experiments

Firstly, the preprocessed ECG signal is filtered by mathematical morphology. The result is shown in Fig. 4.

**Fig. 5:**
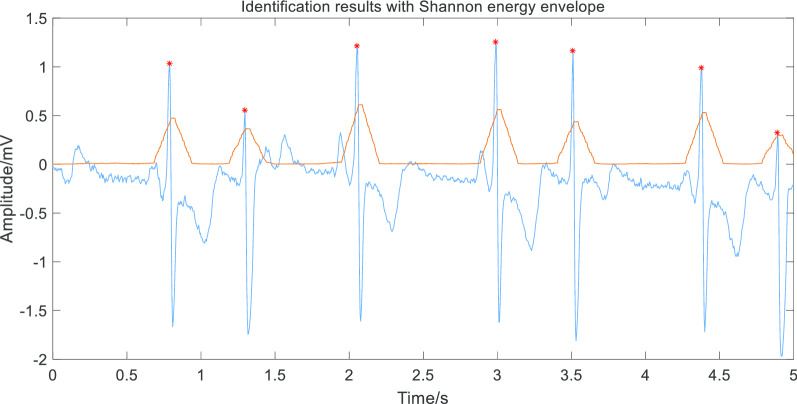
Detecting R wave. The image shows “*” is the result of detection—R peak and indicates the method to be of high accuracy. There is the Shannon energy envelope curve

Then we used Shannon energy calculation for further activation and zero phase shift envelope to extract the envelope curve peak and get R wave position, as shown in Fig. 5.

**Fig. 6:**
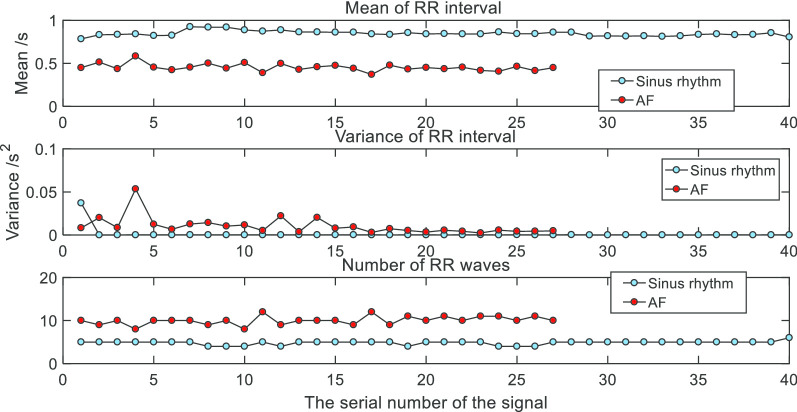
Time-domain features. There are three kinds of features in the image. For the mean of RR interval, sinus rhythm signals were larger than AF signals. For the variance of RR interval, AF ECG signals were a little larger than sinus rhythm signals. For the number of RR intervals, AF signals were more than sinus rhythm signals

After the detection of R waves from sinus rhythm and AF signal segments, we carried on a statistical analysis of mean value, variance and number of R waves of R–R interval. The results were shown in Fig. 6.

### Frequency domain feature extraction

The processes of extracting frequency-domain feature can be divided into three steps: performing four-layer wavelet decomposition, reconstructing based on the fourth layer, performing Fast Fourier Transform and marking the maximum amplitude in the frequency spectrum (MAiFS).

The fourth layer discrete wavelet transform is performed on a single waveform. And we obtained the sub-band signal bandwidth (as shown in Table 1) after decomposition. The sampling frequency is 250 Hz.

**Table 1:** Frequency range of fourth layer discrete wavelet transform

Sub-band	Frequency range ( Hz)
CA4	0–7.813
CD4	7.813–15.625
CD3	15.625–31.25
CD2	31.25–62.5
CD1	62.5–125

The table shows the results of the four-layer discrete wavelet transform

**Fig. 7:**
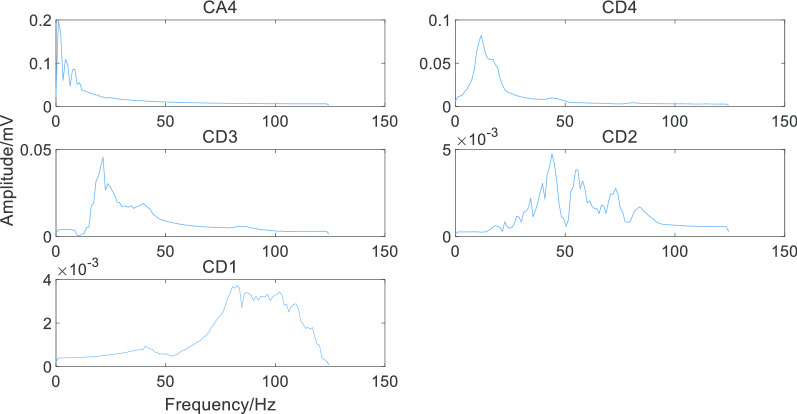
Frequency ranges of sub-band signals. It can be seen that different sub-band has a different spectrum and contains different information in a single waveform

After using the Fast Fourier transform, the sub-band signals of this waveform were shown in Fig. 7 and it could be seen that the frequency distribution of each sub-band signal was consistent with that shown in Table 1.

**Fig. 8:**
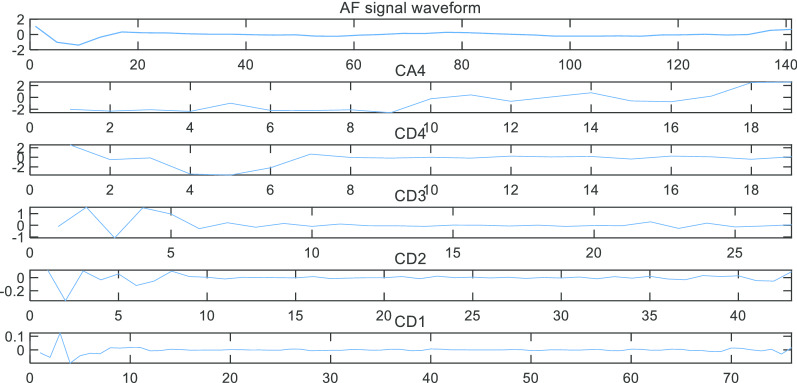
Decomposing single AF signal waveform

**Fig. 9:**
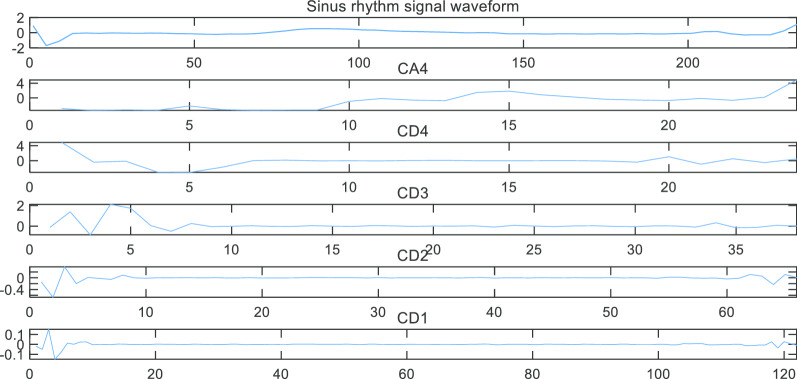
Decomposing single sinus rhythm signal waveform

Then we decomposed the AF signal and sinus rhythm signal according to the frequency range of each sub-band. The results were shown in Figs. 8 and 9.

**Fig. 10:**
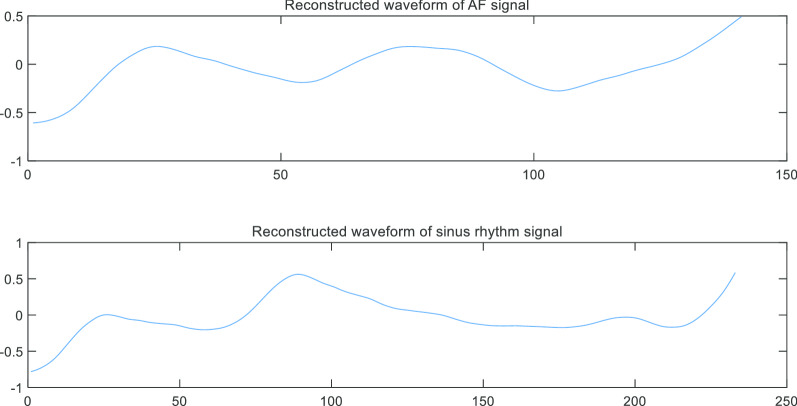
Reconstruction of single AF signal waveform and sinus rhythm signal waveform. The two reconstructed waveforms are largely similar with the extracted single waveforms

Next, we used approximate decomposition coefficients of the fourth layer to reconstruct the sinus rhythm signal and the AF signal. As shown in Fig. 10.

**Fig. 11:**
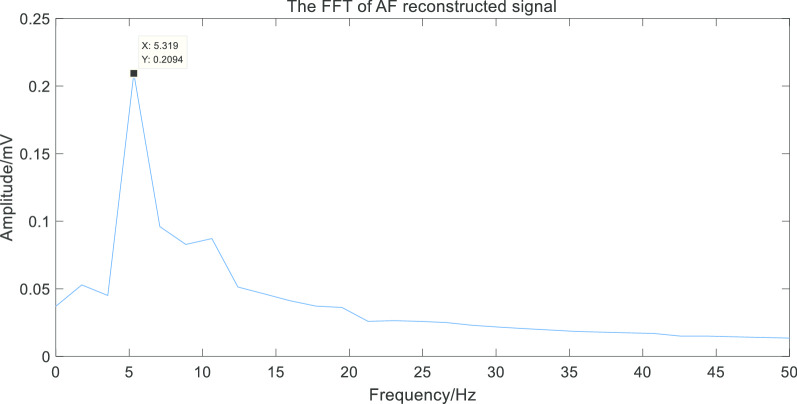
The FFT of AF reconstructed signal

**Fig. 12:**
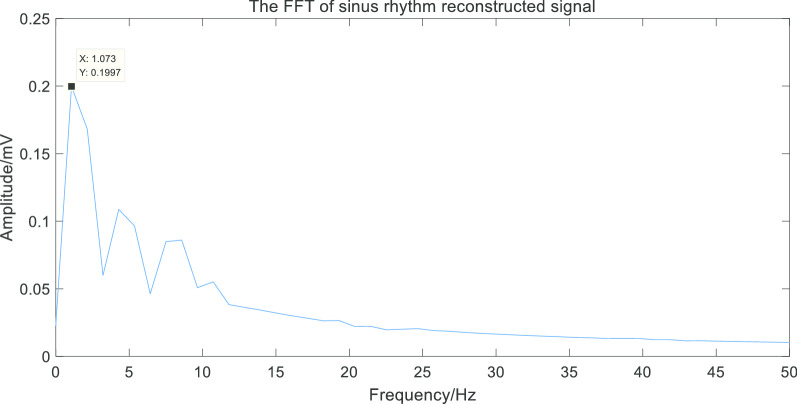
The FFT of sinus rhythm signal

Finally, we performed Fast Fourier transform to analyze the two kinds of the reconstructed signals. As shown in Figs. 11 and 12.

**Fig. 13:**
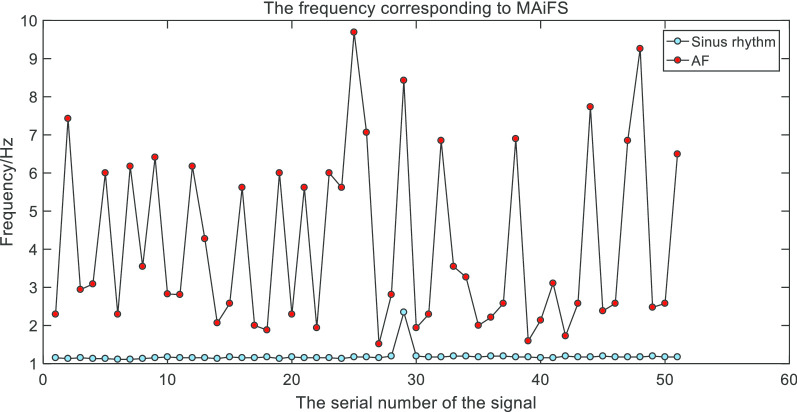
Frequency domain features of AF and sinus rhythm. The frequency-domain feature of AF signals had volatility while sinus rhythm signals had stability

Thus we obtained the frequency corresponding to the maximum amplitude in the spectrum(MAiFS), which can be used as the frequency domain characteristic of the ECG signals. The statistical results of the frequency-domain feature of sinus rhythm signals and AF signals were shown in Fig. 13 (partial data).

#### Classification using decision tree algorithm

**Fig. 14:**
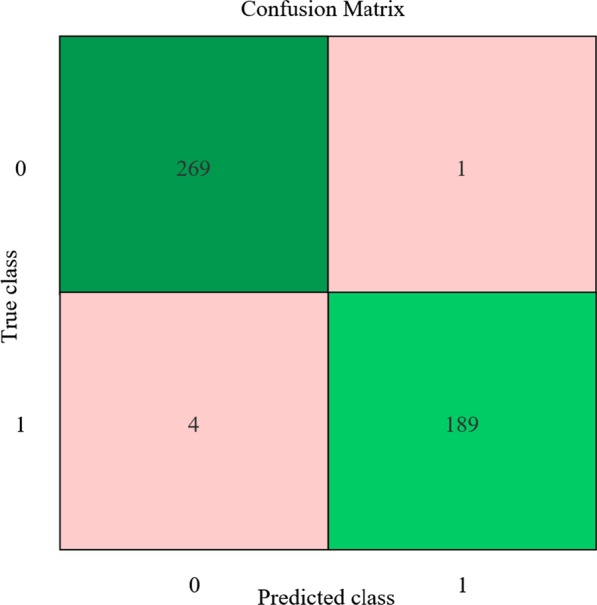
Result of classifying AF signals and sinus rhythm signals. The number in the green rectangle means successful classification and the number in pink rectangle means failed classification

The classifier used a decision tree algorithm [14]. Based on the principle of minimizing the Gini index, a decision tree was generated using the CART (classification and regression tree) algorithm. The data obtained from the above experiments were classified using the generated CART decision tree. And the confusion matrix of the classification results was obtained. As shown in Fig. 14. From the confusion matrix, we knew that the accuracy of classification reaches 98.9%. Sensitivity(SE) and specificity(SP) are calculated as

10 $$SE = \frac{TP}{{TP + FN}}$$

11 $$SP = \frac{TN}{{TN + FP}}$$

where true positive (TP): AF is classified as AF; true negative (TN): sinus rhythm is classified as sinus rhythm; false negative (FN): AF is classified as sinus rhythm; false positive (FP): sinus rhythm is classified as AF. According to the confusion matrix, the sensitivity and specificity of our method were 97.93% and 99.63% respectively. The comparison results were shown in Table 2.

**Table 2:** Comparison with other conclusions

Method	Sensitivity(SE) (%)	Specificity(SP) (%)
Eric Helfenbein et al. [15]	76	97
S Dash et al. [16]	94	95
Tran Thong [17]	89	91
Francisco Rincón [18]	96	93
Proposed algorithm	97.9	99.6

The table shows a comparison with other studies about sensitivity and specificity

### Discussion

Through the extraction of the time-domain feature, we found that sinus rhythm signal and AF signal's R–R interval, the mean of R–R interval, the variance of R–R interval and the number of R waves had significant differences. Therefore, these features could be considered as time-domain features in the ECG signal.

Through the extraction of the frequency-domain feature, we found that the frequency corresponding to the maximum amplitude was intensively located in 1 Hz in the spectrum of the reconstructed sinus rhythm signal. However, in the spectrum of the reconstructed AF signal, the frequency corresponding to the maximum amplitude was discretely located from 2 to 8 Hz, which could be regarded as the dominant frequency in ECG signals with AF. Therefore, the frequency corresponding to the maximum amplitude of the spectrum can be used as the frequency-domain feature to detect AF.

Through the decision tree classification algorithm, we classified the sinus rhythm signals and AF signals with high accuracy. Besides, we also got great sensitivity and specificity compared with other studies.

## Conclusion

The frequency corresponding to the maximum amplitude of the frequency spectrum in the sinus rhythm signal was concentrated and the fluctuation was weak. But the frequency corresponding to the MAiFS in the atrial fibrillation signal is divergent and irregular. Therefore, the experimental results can prove the validity of the frequency corresponding to MAiFS and the practicability and accuracy of the detection method, which applied this frequency-domain feature. Through the detection method, we obtained good accuracy of classifying sinus rhythm signals and AF signals. And the sensitivity and specificity of our method were pretty good by comparison with other studies.

## Data Availability

The MIT-BIH Atrial Fibrillation databases can be found here: https://www.physionet.org/content/afdb/1.0.0/. Accessed 4th Nov 2000.

## Abbreviations

MAiFS: The maximum amplitude in the frequency spectrum
AF: Atrial fibrillation
ECG: Electrocardiogram
